# Ectopic FGFR1 Increases Intracellular Pool of Cholesterol in Prostate Cancer Cells

**DOI:** 10.3390/ijms27031190

**Published:** 2026-01-24

**Authors:** Ziying Liu, Yuepeng Ke, Tingting Hong, Kennedy Smith, Peter Davies, Yun Huang, Dekai Zhang, Sanjukta Chakraborty, Yubin Zhou, Fen Wang

**Affiliations:** 1 Center for Translational Cancer Research, Institute of Biosciences and Technology, Texas A&M Health Science Center, Texas A&M University, Houston, TX 77030, USA; 2 Department of Translational Medical Sciences, Naresh K. Vashisht College of Medicine, Texas A&M University, Houston, TX 77030, USA; 3 Center for Epigenetics and Disease Prevention, Institute of Biosciences and Technology, Texas A&M University, Houston, TX 77030, USA; 4 Center for Infectious and Inflammatory Diseases, Institute of Biosciences and Technology, Texas A&M University, Houston, TX 77030, USA; 5 Department of Medical Physiology, Naresh K. Vashisht College of Medicine, Texas A&M University, Bryan, TX 77807, USA

**Keywords:** prostate cancer, fibroblast growth factor, cholesterol, low-density lipoprotein, sterol regulatory element-binding protein 2

## Abstract

Prostate cancer (PCa) is the most common male cancer and the second leading cause of cancer death in men. Androgen deprivation therapy (ADT) has been widely used as the first-line treatment for PCa. However, most PCa will progress to castration-resistant PCa (CRPC) that resists ADT 1 to 3 years after the treatment. Steroidogenesis from cholesterol is one of the mechanisms leading to ADT resistance. In PCa cells, low-density lipoprotein (LDL) mediated uptake is the major venue to acquire cholesterol. However, the mechanism of regulating this process is not fully understood. Fibroblast growth factor receptor 1 (FGFR1) is a receptor tyrosine kinase (RTK) that is ectopically expressed in PCa cells and promotes PCa progression by activating downstream signaling pathways. To comprehensively determine the roles of FGFR1 in PCa, we generated FGFR1-null DU145 cells and compared the transcriptomes of FGFR1-null and wild-type cells. We found that ablation of FGFR1 reduced the expression of genes promoting LDL uptake and de novo synthesis of cholesterol, thereby reducing the overall cholesterol pool in PCa cells. Detailed mechanistic studies further revealed that FGFR1 boosted the activation of sterol regulatory element-binding protein 2 (SREBP2) through ERK-dependent phosphorylation and cleavage, which, in turn, increased the expression of low-density lipoprotein receptor (LDLR) and enzymes involved in de novo cholesterol synthesis. Furthermore, in silico analyses demonstrated that high expression of FGFR1 was associated with high LDLR expression and clinicopathological features in PCa. Collectively, our data unveiled a previously unrecognized therapeutic avenue for CRPC by targeting FGFR1-driven cholesterol uptake and de novo synthesis.

## 1. Introduction

Prostate cancer (PCa) is the most diagnosed cancer among men in the United States and remains the leading cancer death in 2025 [1]. For those patients with organ-confined, intermediate- to high-grade PCa, the most commonly used treatments are radical prostatectomy, radiotherapy, or androgen deprivation therapy (ADT). However, PCa will ultimately acquire resistance and progress to castration-resistant PCa (CRPC). One mechanism underlying castration resistance is the generation of androgen from cholesterol. Intratumoral androgen levels can be similar to, or even higher than, those in eugonadal men, which leads to ADT resistance [2]. Most cells acquire cholesterol through uptake from the bloodstream, with low-density lipoprotein (LDL) being the major source. LDLs are spherical particles with ~18–29 nm in diameter. They have a central core of cholesterol esters and triglycerides, and an amphipathic shell of phospholipids, free cholesterol, and apolipoprotein B-100 (Apo B-100). LDL binds to the ligand-binding domain of LDL receptor (LDLR) on the cell membrane, which mediates the internalization of LDL and the release of free cholesterol in lysosomes [3].

Expression of LDLR is regulated by the sterol regulatory element-binding protein 2 (SREBP2). SREBP2 is located on the endoplasmic reticulum (ER) membrane, forms a complex with SREBP cleavage-activating protein (SCAP) and insulin-inducible gene 1 (INSIG1). The sterol-sensing domain (SSD) of SCAP monitors cellular cholesterol levels. When cholesterol is sufficient, INSIG will remain associated with SCAP-SREBP2 and keep it inactive [4]. When cholesterol levels are low, INSIG disassociates from SCAP-SREBP2. SREBP2 translocates to the Golgi apparatus, where it undergoes site 1 protease (S1P) and site 2 protease (S2P)-mediated cleavage and phosphorylation to form the nuclear or N-terminal SREBP2 (nSREBP2). The activated nSREBP2 then enters the nucleus, where it promotes gene expression, including LDLR and enzymes in cholesterol de novo synthesis.

The fibroblast growth factor (FGF) signaling pathway contributes to PCa progression and emerges as a potential cancer therapeutic target [5]. The FGF family consists of 22 polypeptides. Four types of FGF receptors (FGFR) mediate FGF functions, designated FGFR1/2/3/4 [6], which transduce downstream signals via activating three canonical pathways: extracellular signal-regulated kinase (ERK), phosphoinositide 3-kinases (PI3K), and phospholipase C γ (PLCγ), as well as several non-canonical downstream signaling pathways. Prostate epithelial cells typically do not express FGFR1. However, PCa cells frequently express FGFR1, and the expression level is significantly associated with short survival time and androgen independence in human PCa [7]. Ectopic expression and constant activation of FGFR1 induced prostate lesions in an expression level-dependent manner [8,9,10]. Ablation of *fgfr1* significantly retards PCa initiation, growth, and metastasis, as well as extends survival time in mouse PCa models [11]. Ectopic FGFR1 expression is associated with castration resistance and PCa progression. Multiple FGFR kinase blocking drugs strongly inhibit PCa growth. It has a long-lasting anti-tumor effect in combination with enzalutamide, the second-generation androgen inhibitor [12]. However, the mechanism by which ectopic FGFR1 regulates cholesterol metabolism in PCa remains unclear.

Therefore, we hypothesized that FGFR1 promotes cholesterol accumulation in PCa and facilitates PCa progression. To test this hypothesis, we first confirmed that FGFR1 expression is positively correlated with intracellular cholesterol levels in PCa cells. Gene set enrichment analysis (GSEA) revealed significant downregulation of cholesterol homeostasis pathways following FGFR1 ablation. We further identified a strong association between FGFR1 and the cholesterol uptake regulator LDLR, and the de novo synthesis rate-limiting enzyme 3-hydroxy-3-methylglutaryl-coenzyme A reductase (HMGCR) at both mRNA and protein levels. Mechanistically, we show that this regulatory axis is mediated through ERK-SREBP2 signaling. Importantly, our work demonstrates translational relevance, as combined inhibition of FGFR and SREBP2 markedly suppresses tumor cell growth.

## 2. Results

### 2.1. PCa Has an FGF-Rich Tumor Microenvironment (TME)

**Figure 1:**
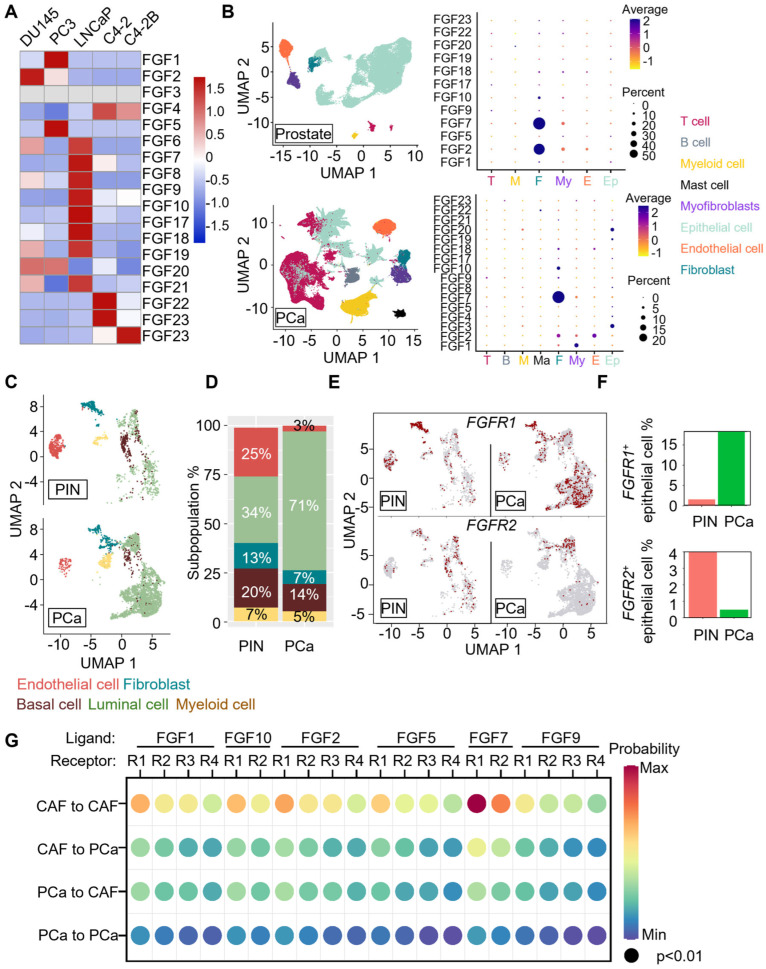
PCa has an FGF-rich microenvironment. (**A**) Quantitative RT-PCR analysis of 18 autocrine/paracrine and endocrine FGFs. The raw data of each gene is normalized to a Z score across five cell lines. The abundance of the mRNA was shown in the heatmap. (**B**) The scRNA-seq dataset (GSE176031, GSE172357, GSE153892, GSE181294, and GSE210358) was downloaded from the GEO database and analyzed with the Seurat pipeline. The data were visualized using uniform manifold approximation and projection (UMAP), which shows the cell composition of the prostate and PCa. The dot plot shows FGF expression levels in each cell population in the prostate and PCa. (**C**) The scRNA-seq dataset (GSE137829) was downloaded from the GEO database and analyzed with the Seurat package in RStudio. The cell compositions in the PIN and PCa groups are presented as UMAPs. (**D**) Quantification of manually identified cell populations shows an increase in the epithelial cell population in the PCa group. (**E**) Expression of FGFR1 and FGFR2 in cell populations. (**F**) Quantitative analyses reveal that compared with the PIN group, the PCa group has an increase in FGFR1^+^ epithelial population and a decrease in FGFR2^+^ epithelial population. (**G**) CellChat analysis of the scRNA-seq dataset used in panel (**B**) reveals the potential FGF/FGFR signaling axes between the cancer-associated fibroblast and cancer cells in human PCa. CAF, cancer-associated fibroblast; PCa, prostate cancer cells; R1–4: fibroblast growth factor receptor 1–4.

To determine the expression levels of FGFs in human PCa cells, we used qPCR to quantify *FGF* expression at the mRNA level in five commonly used PCa cell lines. In DU145 cells, *FGF2*, *FGF19* were highly expressed; in PC3 cells, *FGF1*, *FGF5*, *FGF19* were highly expressed; in LNCaP cells, *FGF6*, *FGF7*, *FGF8*, *FGF9*, *FGF10*, *FGF10*, *FGF18*, *FGF20*, were highly expressed; in C4-2 cells, *FGF4*, *FGF21*, *FGF23* were highly expressed; in C4-2B cells, *FGF4*, *FGF23* were highly expressed (Figure 1A).

To determine whether the FGF expression profile in human PCa was different from that in the healthy prostate, we collected publicly available single-cell RNA-sequencing (scRNA-seq) data from the Gene Expression Omnibus (GEO) database (GSE176031 [13], GSE172357 [14], GSE153892 [15], GSE181294 [16], and GSE210358 [17]), which included 51 human PCa samples (83,376 cells) and 6 normal human prostate tissues (46,390 cells). The expression matrix was converted to a Seurat object using the Seurat package in RStudio and normalized by SCTransform. The batch effect was corrected by Harmony [18]. The results showed that more cancer cells expressed FGFs, including *FGF3*, *FGF4*, *FGF8*, and *FGF21*, than healthy prostate cells. In addition, many epithelial cells in PCa tissues expressed *FGF3* and *FGF20* at an elevated level (Figure 1B), indicating that PCa had an FGF-enriched TME.

To determine FGFR expression in human PCa, we downloaded the publicly available scRNA-seq dataset (GSE137829 [19]) from the GEO database. We compared the FGFR expression profiles in non-tumorous PIN and the primary tumor cells. The gene expression matrix was analyzed with Seurat in RStudio and normalized with log transformation. The data were visualized as UMAP after dimension reduction (Figure 1C). Subsequently, by manual cell identification using the signature genes, the cell populations were quantified and presented as a bar plot (Figure 1D). The luminal cell population was almost doubled in the PCa samples compared to the PIN group. To assess the *FGFR* expression profiles in the PIN and PCa groups, we defined the *FGFR1*^+^ and *FGFR2*^+^ cells by a normalized expression of *FGFR1* and *FGFR2* larger than 0, respectively, and demonstrated them in a UMAP (Figure 1E). In the PCa samples, we observed an increase in *FGFR1*^+^ luminal epithelial cells and a decrease in *FGFR2*^+^ luminal epithelial cells (Figure 1F). The results are in line with our previous finding that the expression of FGFR1 and FGFR2 is mutually exclusive [6].

To identify the FGF/FGFR signaling axis in human PCa, we used CellChat [20] to predict the active FGF/FGFR signaling axis between the cancer-associated fibroblast (CAF) and PCa cells in the human PCa, using the same scRNA-seq datasets as previously described. The interactive probability was visualized by a dot plot. The result showed that in the PCa groups, the predicted interaction of FGF7 from CAF to FGFR1 on CAF was the most significant interaction among all the predicted interactions. Interestingly, the interaction of FGF7 from CAF to FGFR1 on PCa cells was the most significant in PCa cells (Figure 1G). The data further demonstrates the ectopic paracrine interaction between stroma and PCa cells and suggests that the ectopic FGF/FGFR1 interaction in the TME plays an important role in PCa tumorigenesis and progression.

### 2.2. FGFR1 Ablation Downregulates the Expression of Genes Required for Cholesterol Uptake and De Novo Synthesis

**Figure 2:**
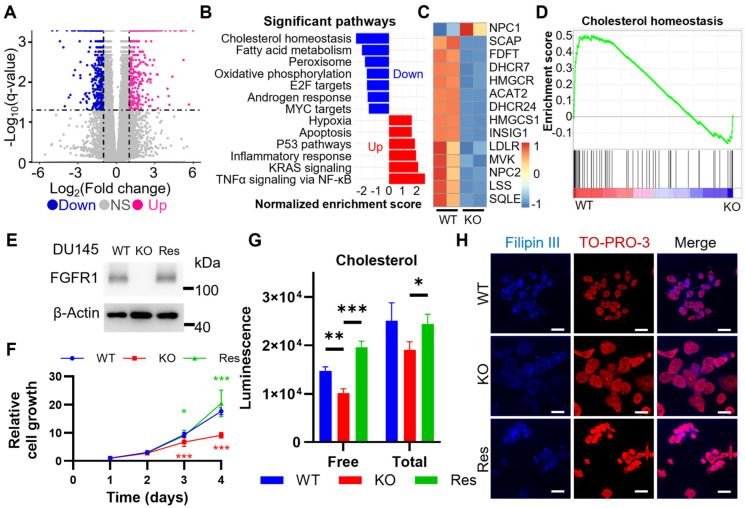
Ablation of FGFR1 perturbs cholesterol homeostasis in DU145 cells. (**A**) Volcano plot reveals that the ablation of FGFR1 changes gene expression profiles in DU145 cells. (**B**) Bar chart of gene ontology (GO) analysis of transcriptome profiles of DU145 and DU145^R1KO^ reveals the most significant pathway changes caused by FGFR1 ablation. (**C**) GSEA plot and (**D**) Heatmap of hallmark genes expression regulating cholesterol homeostasis. (**E**) Western blot confirms FGFR1 expression in the indicated DU145 cells. (**F**) Cell growth curve of the indicated DU145 cells. (**G**) Free and total cholesterol levels in the indicated DU145 cells. (**H**) Filipin III staining shows the total cholesterol levels in the indicated DU145 cells. *: *p* < 0.05; **: *p* < 0.01; ***: *p* < 0.001. WT, wild-type DU145; KO, DU145^R1KO^; Res, DU145^R1Res^. Scale bar, 25 μm.

Ablation of FGFR1 reduces aerobic glycolysis, changes ATP production profiles, retard cell proliferation, and compromises tumorigenicity in DU145 cells that highly express FGFR1 [21]. To determine how FGFR1 signaling evoked such changes in PCa, we used bulk RNA sequencing (RNA-seq) to compare gene expression profiles between wild-type DU145 and FGFR1 null DU145^R1KO^ cells. By setting the threshold to more than two-fold changes both in increase and decrease, and a *q* value less than 0.05, we identified 1620 differentially expressed genes (DEG). Among them, 717 genes were upregulated, and 903 genes were downregulated (Figure 2A).

To better understand how gene expression profile changes were attributed to phenotypic changes, we performed gene ontology analysis to identify the alterations in pathways enriched among genes. It was clear that the molecules involved in cholesterol homeostasis were the most significantly altered pathway in DU145^R1KO^ cells (Figure 2B). The GSEA enriches the ranked genes and demonstrates that the expression of most genes regulating cholesterol homeostasis was downregulated in FGFR1 null DU145^R1KO^ cells (Figure 2C,D). Among these genes were Niemann–Pick disease, types C1 and C2 (NPC1 and NPC2), LDLR, SCAP, and 3-hydroxy-3-methylglutaryl-CoA synthase 1 (HMGCS1).

To validate the findings from RNA-seq data that FGFR1 promoted cholesterol uptakes and de novo synthesis, we first performed a bioluminescence assay to compare free cholesterol levels in DU145^R1KO^ cells with those in wild-type DU145 cells (Figure 2E). It was clear that the free cholesterol levels were significantly lower in FGFR1 null DU145^R1KO^ cells than in wild-type DU145 cells. By treating the cells with cholesterol esterase that hydrolyzes cholesterol esters to free cholesterol, we furthermore assessed total cholesterol pools in the cells. The data revealed that the ablation of FGFR1 in DU145 cells also lowered total cholesterol in the cells (Figure 2G). Confocal microscopic analyses with Filipin III staining also revealed a significant reduction in free cholesterol in FGFR1 null DU145^R1KO^ cells (Figure 2H). Thus, the results confirmed that the disruption of FGFR1 signaling reduced cholesterol in PCa cells.

To determine that the reduction in cholesterol was indeed FGFR1 kinase-dependent, we reinstated FGFR1 expression in DU145^R1KO^ cells, designated DU145^R1Res^ (Figure 2E). Reinstating FGFR1 expression in DU145 cells rescued the growth retardation caused by FGFR1 ablation (Figure 2F). As predicted, reinstating FGFR1 expression also significantly increased free and total cholesterol in DU145^R1Res^ cells to a level comparable to wild-type DU145 cells (Figure 2G,H). These results indicate that FGFR1 signaling augments the cholesterol pool in PCa cells.

### 2.3. FGFR1 Promotes LDL Uptake in PCa Cells

**Figure 3:**
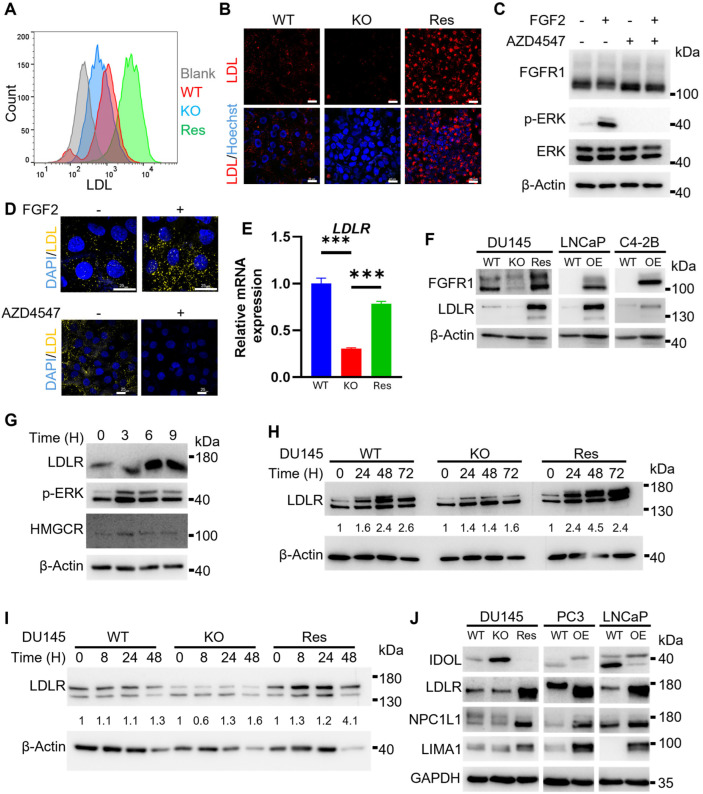
FGFR1 increases cholesterol uptake and LDLR expression. (**A**) Flow cytometry quantification of fluorescently labeled LDL uptake in the indicated DU145 cells. (**B**) Live-cell imaging of fluorescently labeled LDL uptake in DU145 cells. (**C**) Western blot analysis confirming FGFR1 activation by FGF2 and inhibition by AZD4547 in DU145 cells. (**D**) Confocal images showing increased LDL uptake in DU145 WT cells following FGF2 treatment and reduced uptake after AZD4547 treatment. (**E**) qPCR and (**F**) Western blot analyses showing a positive correlation between FGFR1 and LDLR expressions. (**G**) Western blot analysis showing expressions of LDLR and HMGCR are upregulated in DU145 WT cells treated with 20 ng/mL FGF2 for the indicated time. (**H**) Western blot analysis shows LDLR protein levels in the indicated DU145 cells treated with 10 μM chloroquine for 24–72 h. (**I**) Western blot analysis of LDLR protein in the indicated DU145 cells treated with 10 μg/mL cycloheximide for 8–48 h. (**J**) Western blot analysis of LDLR, IDOL, NPC1L1, and LIMA1 expression in PCa cells. ***: *p* < 0.001. WT, wild-type; KO, DU145^R1KO^; Res, DU145^R1Res^; OE, FGFR1 overexpression. Scale bar: 25 μm.

Since most cells acquire cholesterol primarily through LDL-mediated uptake, we pretreated DU145 cells with fluorescence-labeled LDL 2 h prior to the incubation with fluorescence-labeled LDL. The cells were harvested for flow cytometry analyses (Figure 3A). The results clearly showed that LDL uptake was noticeably lower in FGFR1 null DU145^R1KO^ cells compared with wild-type DU145 cells. As expected, LDL uptake in DU145^R1Res^ was significantly increased. Confocal microscopy imaging confirmed the changes in cholesterol uptake among these cells (Figure 3B). The results indicate that FGFR1 promotes LDL uptake in DU145 cells.

Additionally, to determine whether the activity of FGFR1 to promote cholesterol uptake was ligand-dependent, DU145 cells were treated with FGF2 at a concentration of 20 ng/mL to activate FGFR1 kinase prior to the incubation with fluorescence-labeled LDL. Western blot analyses revealed that FGF induced ERK phosphorylation, a downstream target of the FGFR1 signaling pathway (Figure 3C). Confocal microscopy imaging showed that FGF2 enhanced cholesterol uptake, and the activity diminished when the cells were treated with AZD4547, an FGFR kinase inhibitor (Figure 3D). The results indicate that FGFR1 promotes cholesterol production in a ligand-dependent and kinase-activity-dependent manner.

LDLR is the major transporter that mediates LDL uptake. To determine the mechanism by which FGFR1 promoted LDL uptake in PCa cells, we assessed the expression of *LDLR* in DU145 cells by quantitative real-time RT-PCR. The result showed that the expression of LDLR was downregulated in FGFR1 null DU145^R1KO^ cells. However, the expression was increased in DU145^R1Res^ cells to a level similar to wild-type DU145 cells, suggesting that reinstatement of FGFR1 restored LDLR expression in the cells (Figure 3E). To validate the finding that FGFR1 upregulated LDLR expression at the protein level, we extracted proteins from DU145 cells with or without FGFR1 expression and measured LDLR proteins with a Western blot. The results showed that the expression of LDLR was reduced in DU145^R1KO^ and increased in DU145^R1Res^ cells (Figure 3F).

To determine whether exogenous FGF enhanced FGFR1 activity to promote LDLR expression, we treated DU145 cells with FGF2 at a concentration of 10 ng/mL. The cells were then lysed at 0, 3, 6, and 9 h after the treatment and subjected to Western blot analyses for LDLR expression. The results showed that LDLR expression was increased in a time-dependent manner (Figure 3G). The results demonstrate that the activation of LDLR by FGFR1 is ligand-dependent.

To determine how FGFR1 promoted LDLR protein expression in PCa cells, we treated DU145 cells with chloroquine (a lysosome inhibitor) that blocks lysosome-mediated protein degradation. 24–72 h after the treatment, the cells were lysed and subjected to Western blot analyses. The data showed that compared to wild-type DU145 cells, LDLR accumulations in DU145^R1KO^ cells were significantly lower after the chloroquine treatment (Figure 3H). In contrast, LDLR accumulations in DU145^R1Res^ were higher than those in wild-type and FGFR1 null DU145 cells. On the other hand, treating cells with cycloheximide, a protein synthesis inhibitor, did not affect LDLR protein expression (Figure 3I). Together, the results reveal that FGFR1 promotes LDLR expression at both transcription and translation levels.

To determine whether FGFR1 also regulated LDLR expression in other PCa cells, we overexpressed FGFR1 in LNCaP and C4-2B cells since the cells expressed FGFR1 at a low level, designated LNCaP^R1OE^ and C4-2B^R1OE^, respectively (Figure 3F). As expected, the expression of LDLR was significantly increased in LNCaP^R1OE^ and C4-2B^R1OE^ cells from the parental wild-type cells.

Niemann-Pick C1-like intracellular cholesterol transporter 1 (NPC1L1) and LIM domain and actin-binding protein 1 (LIMA1) are key carriers for cholesterol intracellular transportation [22,23]. To determine whether FGFR1 also regulated the expression of NPC1L1 and LIMA1, we assessed their expression in DU145, PC-3, and LNCaP cells with or without FGFR1 expression. The result showed that NPC1L1 expression decreased in FGFR1 null in DU145^R1KO^ cells and was restored in DU145^R1Res^ cells to a level comparable to wild-type DU145 cells (Figure 3J). Consistently, overexpression of FGFR1 also increased NPC1L1 and LIMA1 expression in PC-3 and LNCaP cells. Interestingly, the expression of the inducible degrader of the LDLR, which targeted LDLR for degradation, was also regulated by FGFR1 in all three cell lines. The data suggested that FGFR1 also promotes cholesterol intracellular transportation and, therefore, cholesterol content in PCa cells.

**Figure 4:**
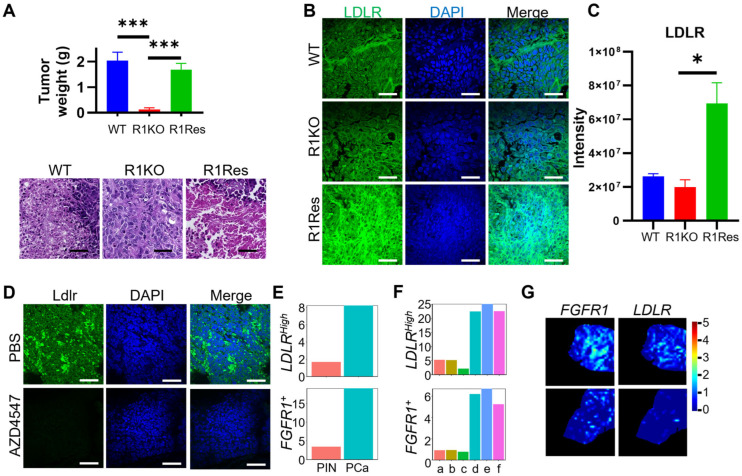
FGFR1 enhances LDLR expression in vivo. (**A**) Bar plot of xenograft weights and H&E staining of xenografts (*n* ≥ 2). (**B**) Confocal images of the indicated xenograft sections immunostained with the anti-LDLR antibody. (**C**) Quantification of LDLR intensity in DU145 xenograft sections. (**D**) Ldlr expression is decreased in TRAMP tumors treated with the FGFR inhibitor AZD4547 (*n* = 3). (**E**) Quantification of FGFR1^+^ and LDLR^high^ epithelial cell ratios in patient samples in the GSE137829 dataset downloaded from the GEO database. (**F**) Quantification of FGFR1^+^ and LDLR^high^ epithelial cell ratio in patient samples in the GSE176031 dataset downloaded from GEO database. a–f: samples a–f. (**G**) Bioinformatic analysis of the spatial transcriptomics data from STProstate Research [24] database shows that FGFR1 and LDLR expressions are overlapped in tumor tissues. Scale bar: 50 μm. *: *p* < 0.05. ***: *p* < 0.001. WT, wild-type; KO, DU145^R1KO^; Res, DU145^R1Res^.

Next, to determine whether FGFR1 regulated LDLR expression in a tumor setting, we implanted wild-type DU145, DU145^R1KO^, and DU145^R1Res^ cells subcutaneously in the flanks of immunodeficient mice. Consistent with our previous reports [21], the xenograft derived from DU145^R1KO^ cells was significantly smaller than the wild-type control. The xenograft derived from DU145^R1Res^ cells was comparable to that of wild-type, suggesting that reinstating FGFR1 expression restored the tumorigenicity in DU145 cells. H&E staining revealed no significant changes in tumor histology (Figure 4A). We then employed immunostaining with anti-LDLR antibody to assess LDLR expression. It was clear that LDLR expression was reduced in DU145^R1KO^ cells and increased in DU145^R1Res^ cells (Figure 4B,C).

To determine whether FGFR kinase activity was required for promoting LDLR expression in sporadic PCa in the TRAMP model, we treated the tumor-bearing TRAMP mice with AZD4547, an FGFR inhibitor, through intraperitoneal injection for two weeks. The results showed that the AZD4547 treatment significantly reduced Ldlr expression in the tumor (Figure 4D).

To determine whether expression of LDLR was associated with FGFR1 expression at the mRNA level in human PCa, we downloaded scRNA-seq datasets (GSE176031 [13], GSE137829 [14], GSE176031 [13], GSE137829 [14]) from the GEO database and accessed the spatial-transcriptomic datasets (STProstateResearch [24]). Bioinformatic analyses of both scRNA-seq and spatial transcriptomic datasets revealed that LDLR expression in FGFR1-positive epithelial cells was higher than in FGFR1-negative cells (Figure 4E,F). In addition, the FGFR1-expressing cells overlapped with those expressing LDLR (Figure 4G). Together, these data revealed that LDLR expression is associated with FGFR1 expression in human PCa.

### 2.4. FGFR1 Promotes the Activation of SREBP2, a Transcription Factor That Controls LDLR Expression, Through the ERK-Mediated Pathway

**Figure 5:**
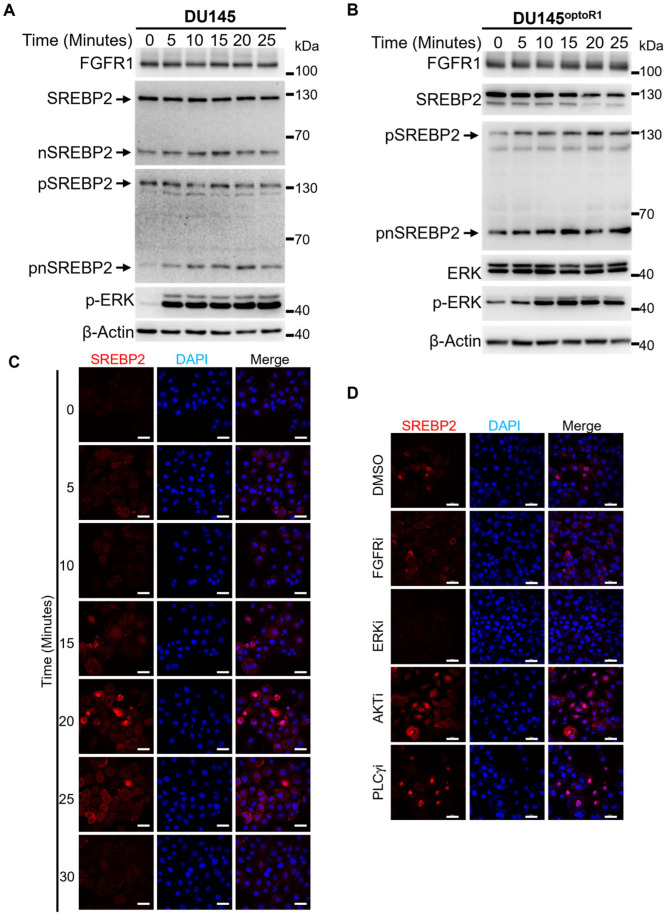
FGFR1 activates SREBP2 through the ERK pathway. (**A**) Stimulation of DU145 cells with 20 ng/mL FGF2 increases SREBP2 phosphorylation and cleavage. (**B**) Activation of optoFGFR1 in DU145^optoR1^ cells with blue light induces activation of SREBP2. (**C**) DU145 cells were treated with 20 ng/mL FGF2 for the indicated times, and the cellular localization of SREBP2 was examined using confocal microscopy. (**D**) DU145 cells were pretreated with inhibitors for AKT (LY294002, 10 μM), ERK1/2 (SL327, 10 μM), PLCγ (U73122, 2 μM), and FGFR1 (AZD4547, 100 nM) for 24 h, followed by stimulation with 20 ng/mL FGF2. The cellular localization of SREBP2 was visualized with confocal microscopy. DAPI was used for nuclear counterstaining. β-actin was used for internal loading controls. Scale bar, 50 μm. SREBP2, sterol regulatory element-binding protein 2; pSREBP2, phosphorylated SREBP2; nSREBP2, cleaved N-terminal domain of SREBP2; pnSREBP2, phosphorylated nSREBP2.

SREBP2 is a transcription factor regulating LDLR expression. It can be activated through protease cleavage at the Leucine 484-Cysteine 485 bond [25] and phosphorylation on serine 455 [26]. To determine whether FGFR1 promoted SREBP2 cleavage and phosphorylation, DU145 cells were treated with FGF2 at a concentration of 20 ng/mL for 5–25 min as indicated. The cells were then lysed, and the cell lysates were subjected to Western blot analyses for the abundance of phosphorylated SREBP2 (pSREBP2) with anti-pSREBP2 antibodies. The activation of the FGFR1 pathway was confirmed by phosphorylation of ERK (Figure 5A). We observed a significant increase in nSREBP2 and phosphorylated nSREBP2 (pnSREBP2) bands in a ligand-dependent manner (Figure 5A), suggesting that the activation of FGFR1 promoted SREBP2 phosphorylation and cleavage. Note that the full-length phosphorylated SREBP2 was reduced due to the cleavage.

To confirm that the activation of SREBP2 was FGFR1 tyrosine kinase-dependent, we generated DU145 cells carrying a blue light-activatable FGFR1(optoFGFR1), as previously described [27], designated DU145^optoR1^. When DU145^optoR1^ cells were treated with blue light, both nSREBP2 and pSREBP2 were increased in a time-dependent manner, and the full-length (non-cleaved) SREBP2 decreased simultaneously (Figure 5B). The data further provide evidence that the activation of FGFR1 leads to SREBP2 phosphorylation and cleavage.

To determine whether FGFR1 promoted SREBP2 nuclear translocation, we treated the DU145 cells with FGF2 at a concentration of 20 ng/mL and assessed SREBP2 localization from 0 to 30 min after the treatment by the co-localization of SREBP2 and DAPI staining. Clearly, the intensity of nuclear-localized SREBP2 was increased from 15 to 20 min after FGF2 treatment, suggesting that the activation of FGFR1 promoted SREBP2 nuclear translocation (Figure 5C). Notably, the fluorescence intensity of nuclear SREBP2 decreased after 25 min after the treatment, which was likely due to the ubiquitin-mediated degradation previously reported [4,28].

To determine whether the three canonical downstream pathways, ERK, PLCγ, and PI3K/AKT, were required for SREBP2 nuclear translocation, we treated DU145 cells with inhibitors targeting these three pathways. The results showed that the FGF2-induced SREBP2 nuclear translocation was diminished in the ERK inhibitor and FGFR inhibitor treatment groups, but not in the AKT and PLCγ inhibitor groups (Figure 5D). The results indicate that the ERK pathway mediates the activity of FGFR1 to promote SPREBP2 activation.

### 2.5. FGFR1 Promotes the Expression of Cholesterol De Novo Synthesis Enzymes and Downregulates Cholesterol Efflux in PCa

**Figure 6:**
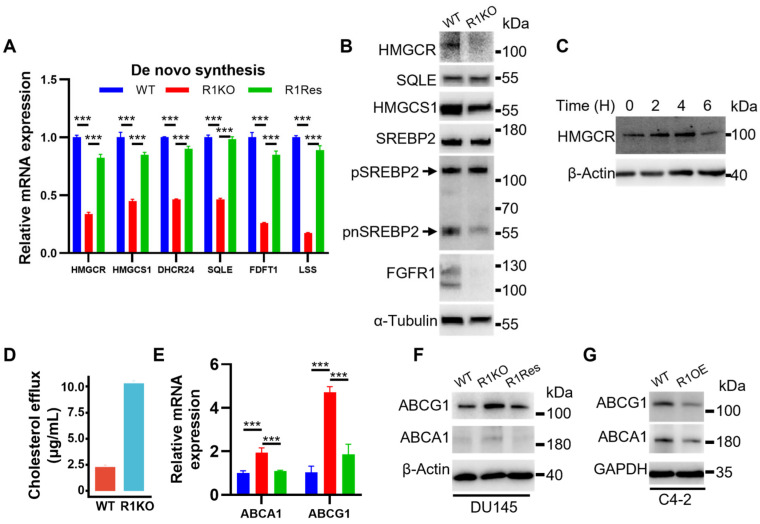
Ectopic FGFR1 enhances cholesterol biosynthesis and reduces cholesterol efflux. (**A**) qRT-PCR quantification and (**B**) Western blot analyses showing expression of key enzymes in cholesterol de novo synthesis in the indicated DU145 cells. (**C**) Western blot analysis of HMGCR expression in DU145^optoR1^ cells after 2 to 6 h of blue light stimulation. (**D**) Cholesterol efflux analysis in DU145 cells. (**E**–**G**) qRT-PCR and Western blot analyses show expression of ABCA1 and ABCG1 in the indicated PCa cells. ***: *p* < 0.001. WT, wild-type; KO, DU145^R1KO^; Res, DU145^R1res^; OE, FGFR1 overexpression. β-actin, α-Tubulin, and GAPDH were used as internal loading controls.

The transcriptomic analyses revealed that the expression of multiple enzymes in the cholesterol de novo synthesis was significantly reduced in FGFR1 null DU145^R1KO^ cells. Among them was HMGCR, a rate-limiting enzyme in the cholesterol de novo synthesis (Figure 2C). To validate this result, we employed quantitative real-time RT-PCR analysis to compare the expression of these enzymes in DU145, DU145^R1KO^, and DU145^R1Res^ cells. The result confirmed a significant decrease in the expression of these enzymes in DU145^R1KO^ cells at the mRNA level (Figure 6A). However, the expression of these enzymes was restored to a level comparable to wild-type DU145 cells. In addition, Western blot analyses confirmed the reduction in these enzymes in DU145^R1KO^ cells at the protein level (Figure 6B). The results indicated that FGFR1 promotes the expression of enzymes in the de novo synthesis of cholesterol and therefore increases cholesterol levels in PCa cells. Consistently, activating optoFGFR1 with blue light also elevated HMGCR expression in DU145^optoR1^ cells at the protein level (Figure 6C).

The homeostasis of cellular cholesterol is maintained through de novo synthesis, uptake, and efflux. To determine whether ablation of FGFR1 affected cholesterol efflux in PCa cells, we measured cholesterol efflux in DU145^R1KO^ cells. The result revealed a significant increase in cholesterol efflux in the FGFR1 null DU145^R1KO^ cells compared to those in wild-type DU145 cells (Figure 6D). To determine whether expressions of the key transporters for cholesterol efflux were regulated by FGFR1, we performed real-time RT-PCR and Western blot to compare the expressions of ATP-binding cassette subfamily A member 1 (ABCA1) and ATP-binding cassette subfamily G member 1 (ABCG1) in DU145^R1KO^ and wild-type DU145 cells. The results showed an increase in the ABCG1 and ABCA1 expressions in DU145^R1KO^ cells compared to wild-type DU145 cells (Figure 6E,F). As expected, reinstating FGFR1 expression lowered the expression of ABCA1 and ABCG1 to a level comparable to that in wild-type DU145 cells. Similarly, the expression of ABCA1 and ABCG1 was also reduced in FGFR1 overexpressing C4-2B cells (Figure 6G). Together, the results suggest that FGFR1 suppresses ABCA1 and ABCG1 expression, and therefore cholesterol efflux in PCa cells.

### 2.6. Cotreating PCa Cells with FGFR1 and SREBP2 Inhibitors Synergistically Suppresses PCa Cell Growth

**Figure 7:**
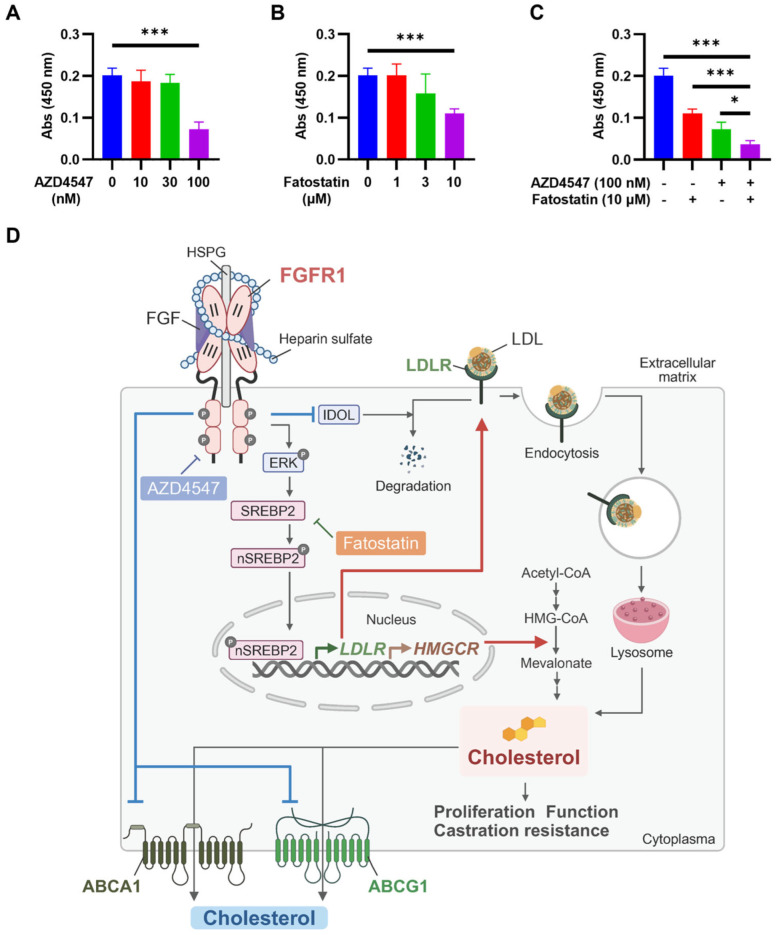
Suppressing FGFR1 kinase potentiates cell growth arrest due to SREBP2 inhibition. (**A**–**C**) DU145 cells were treated with AZD4547, Fatostatin, or 100 nM AZD4547/10 μM Fatostatin for 3 days. The viable cells were stained with the CCK-8 and quantified with a plate reader. (**D**). FGFR kinase and SREBP2 inhibitors synergistically reduce intracellular cholesterol pools and suppress cell growth in PCa cells. FGFR1 activates SREBP2 via ERK signaling, leading to SREBP2 phosphorylation and cleavage. The activated SREBP2 is translocated to the nucleus and enhances LDLR and HMGCR expression, thereby promoting LDLR-mediated cholesterol uptake and de novo biosynthesis. FGFR1 also suppresses IDOL (an E3 ubiquitin ligase that regulates LDLR degradation) expression and hence increases LDLR accumulation, as well as the expression of two cholesterol efflux transporters, ABCA1 and ABCG1, and thereby reduces cholesterol efflux. Suppression of FGFR1 and SRPB2 synergistically reduces intracellular cholesterol pools needed for cell growth, function, and castration resistance. Created in BioRender. Ziying Liu. (2026), https://BioRender.com/zsmvv9n (accessed on 21 January. 2026). Figure created with BioRender.com. *: *p* < 0.05; ***: *p* < 0.001.

As a major component of plasma membranes, cholesterol contributes to membrane integrity and fluidity and, therefore, is important for cell growth, viability, and function. It also serves as a precursor for steroid hormones. To determine whether inhibiting FGFR1 kinase augmented the effect of cholesterol de novo synthesis on DU145 cell growth, DU145 cells were treated with the FGFR1 inhibitor AZD4547 combined with the SREBP2 inhibitor Fatostatin. The results showed that AZD4547 and Fatostatin suppressed DU145 growth at a concentration of 100 nM and 3 μM, respectively (Figure 7A,B). More importantly, cotreatment with ADZ4547 and Fatostatin exhibited stronger activity in suppressing DU145 cell growth than each individual treatment (Figure 7C). The results suggest that the FGFR and SREBP2 inhibitors elicit synergistic inhibitory effects on PCa cell growth, although more thorough and rigorous studies, both in vitro and in vivo, are needed to assess the translational value of double inhibition of FGFR tyrosine kinase and SREBP2 for CRPC treatment.

## 3. Discussion

FGF and FGFR expressions are highly spatiotemporally specific. Each FGFR isoform exerts both common and receptor-specific signals. Perturbations in FGF and FGFR expression are frequently associated with cancers [6,29,30]. It has been demonstrated that ectopic FGFR1 in PCa cells promotes aerobic glycolysis while suppressing OXPHOS, stabilizes IRP2 to increase TFR1 expression, promotes iron accumulation [31], and enhances the expression of choline kinase α (CHKA), thereby increasing phosphocholine [32]. Furthermore, FGFR1 promotes the expression of cyclooxygenase-2 (COX2) and F4/80 and augments NF-κB signaling, demonstrating that FGFR1 promotes chronic inflammation and immunosuppression in the TME [21,33].

Herein, we utilized bulk RNA-sequencing and discovered that the genes regulating the intracellular cholesterol pool were the most significant targets of ectopic FGFR1 in PCa cells. Free and total cholesterols were elevated in PCa cells that highly expressed FGFR1 and were downregulated by FGFR1 ablation. Mechanistically, FGFR1 increased LDLR expression at the mRNA level through the ERK pathway-mediated activation of SREBP2, a transcription factor for LDLR transcription, as well as at the protein level through inhibiting degradation. Inhibition of the other two downstream pathways, PLCγ or PI3K, did not block SREBP2 nuclear translocation and instead resulted in enhanced SREBP2 nuclear translocation, likely due to feedback compensation for the loss of signals mediated by the two pathways. These mechanisms are well recognized as features of oncogenic kinase signaling networks and a known limitation of signal pathway inhibition in cancer [34].

In addition, FGFR1 promoted the expression of enzymes for the cholesterol de novo synthesis, including the rate-limiting enzyme HMGCR, as well as suppressed the expression of two cholesterol efflux transporters and thereby reduced cholesterol discharges. Taken together, the results demonstrate that FGF signaling increases cholesterol pools in PCa cells via multiple mechanisms (Figure 7D). Cholesterol is a building block for membranes and is required for cell integrity, growth, and survival. It also serves as a precursor for steroid hormones, including testosterone, aldosterone, and glucocorticoid hormones, and therefore contributes to ADT resistance. Previous studies report that FGF can activate AR and AR target genes in the absence of androgen [35]. Our findings reveal a novel mechanism by which ectopic FGFR1 promotes PCa growth and progression, which can serve as a new therapeutic target for CRPC treatment.

The cell membrane is primarily composed of a phospholipid bilayer, with proteins, cholesterol, and carbohydrates embedded or attached. About half of the lipids are phospholipids and glycolipids. The other major component of the cell membrane is cholesterol. Cholesterol is inserted into the phospholipid bilayer, maintaining rigidity and fluidity of the membrane. Cancer cells have a high level of intracellular cholesterol that serves as a source of building blocks for their cell membrane [36]. It is consistent with our results that FGFR1 elevates intracellular cholesterol and enables the cells to undergo fast proliferation.

Cholesterol de novo synthesis mainly occurs in the liver [4,37]. In the liver, free cholesterol is converted to cholesterol ester (CE) by acetyl-CoA acetyl transferase (ACAT) in the ER and is packaged with apolipoprotein B (Apo-B) and triacylglycerol to form very low-density lipoprotein (VLDL) through microsomal triglyceride transfer protein (MTP)-mediated lipidation. The nascent VLDL is then transported to the Golgi for glycosylation and secreted into the circulation through exocytosis. Lipoprotein lipase (LPL) hydrolyses and converts VLDL to intermediate density lipoprotein (IDL) and LDL. LDL uptake is mediated by the clathrin-coated vesicles through endocytosis, transporting the LDL-LDLR complex to the lysosome, and releasing free cholesterol through hydrolysis by lysosomal acid lipase (LAL). The NPC1 and NPC2 are two lysosomal proteins that mediate the export from the lysosome. NPC2 is soluble in the lysosome and capable of binding to the free cholesterol in the lysosome lumen and transferring cholesterol to NPC1. NPC1 is located on the lysosomal and endosomal membrane. Cholesterol binds to the NPC1 N-terminal luminal domain (NTD) and transmembrane domain and is eventually released from the lysosome [38]. Free cholesterol is transported to the plasma membrane through the short form of oxysterol-binding protein-related protein (ORP1S), as well as to the ER through the long form ORP1L. On the plasma membrane, there are three pools of cholesterol: accessible cholesterol, the sphingomyelin sequestered pool, and the residual pool. GRAM domain-containing 1 (GRAMD1) on the ER can sense the transient expansion of the accessible cholesterol pool on the PM via its GRAM domain and then promote transport to the ER through the StART-like domain [3]. Herein, we showed that ablation of FGFR1 in DU145 cells reduced LDL uptake and decreased the colocalization of LDL with lysosomes. Conversely, forced expression of FGFR1 in PCa cells increased LDL uptake and de novo synthesis in an FGFR1 kinase-dependent manner. Taken together, these findings suggest that FGFR1 increases intracellular cholesterol in PCa cells.

Other than SREBP2, another regulator of cholesterol homeostasis is the liver X receptor (LXR) pathway. While SREBP2 mainly regulates cholesterol accumulation, LXR mainly regulates the removal of cholesterol [3,4]. The free cholesterol can be excreted through ATP-binding cassette transporter A1 (ABCA1) and ATP-binding cassette subfamily G member 1 ABCG, whose expression is regulated by LXR [4]. Our data showed that disruption of FGFR1 signaling enhanced the expression of ABCA1 and ABCG1 and increased the efflux of cholesterol from the PCa cells. In addition to SREBP2, FGFR1 may also inactivate LXR via the ERK-mediated pathway [39]. Therefore, disruption of the FGFR1-ERK pathway activates LXR, induces ABCA1 expression, and increases cholesterol efflux.

Cholesterol serves as the precursor for androgen biosynthesis and supports intratumoral steroidogenesis. The clinically used drug abiraterone acetate suppresses steroidogenesis but typically induces resistance after 9 to 15 months [40]. Our findings suggest that depleting cholesterol in PCa cells by inhibiting FGFR1 may attenuate steroidogenesis and thereby impede PCa progression. Moreover, advanced PCa frequently metastasizes to distant organs such as the bone, liver, lungs, and lymph nodes. Elevated cholesterol levels have been reported to promote the epithelial-to-mesenchymal transition (EMT) [41], enhance cell motility, and facilitate metastasis of PCa. Consistently, our laboratory previously demonstrated that ablation of FGFR1 upregulates the epithelial marker E-cadherin while downregulating the mesenchymal marker vimentin in PCa cells [32]. These observations collectively support the role of FGFR1 in promoting PCa metastasis, and our research further elucidates this regulation from a cancer metabolism perspective. Additionally, cancer cells often remodel the plasma membrane to develop drug resistance. The tumor microenvironment is typically acidic due to hypoxia and aerobic glycolysis, which neutralizes the negatively charged lipids, alters lipid organization, and compacts the membrane structure, ultimately reducing drug permeability [42]. Our results show that blocking SREBP2 activation using Fatostatin, in combination with the FGFR inhibitor AZD4547, significantly reduces tumor growth and potentially enhances the penetration of anti-tumor compounds. In addition, further analysis of cholesterol metabolism in PCa and other diseases is essential for a systematic understanding of tumor treatment mechanisms. Hepatic cholesterol metabolism may also influence tumor lipid availability and therapeutic responses [43,44].

## 4. Materials and Methods

### 4.1. Cell Culture and Viability Assay

Human PCa cells DU145, PC3, and LNCaP, and the lentiviral packaging cells Lenti-X^TM^ 293T cells (TaKaRa, San Jose, CA, USA) were cultured in Dulbecco’s Modified Eagle’s Medium (DMEM) (GenDEPOT, Baker, TX, USA). Human C4-2 and C4-2B PCa cells were cultured in Roswell Park Memorial Institute medium (RPMI) 1640 (GenDEPOT, Baker, TX, USA). Both media were supplemented with 10% fetal bovine serum (FBS) (GenDEPOT, Baker, TX, USA) and 1% penicillin–streptomycin (PS) solution (GenDEPOT, Baker, TX, USA). The cells were cultured at 37 °C with 5% CO_2_. For quantitation assays, the PCa cells were seeded in 96-well plates at a density of 500 cells per well in 100 μL complete medium. Inhibitors were added to the medium as needed, 24 h after seeding. In total, 10 μL of the cell counting kit-8 (CCK-8) solution (DOJINDO, Rockville, MD, USA) was added to each well on the day of the assay. After incubating at 37 °C with 5% CO_2_ for 2 h, the absorbance at 450 nm was measured by a Cytation 5 Cell Imaging Multimode Reader (Agilent/BioTek, Santa Clara, CA, USA).

### 4.2. Flow Cytometry

The cells were stained with pHrodo™ Red LDL (Thermo Fisher Scientific, Waltham, MA, USA) for 2 h. The cells were then digested with trypsin, spun down, and resuspended in the flow cytometry buffer (5% FBS and 1% BSA in PBS). Next, the cells were passed through a 35 μm cell strainer and collected in a round-bottom test tube (Corning, Corning, NY, USA). The intensity of pHrodo Red LDL was measured with an LSR II flow cytometer (BD Bioscience, Franklin Lakes, NJ, USA). The raw data were analyzed with FlowJo software (v10).

### 4.3. Cholesterol Assay

For free cholesterol assays, the cells were seeded in a 6-well dish at a density of 0.3 million cells per well with 2 mL of complete medium. The cholesterol was measured with the Cholesterol/Cholesterol Ester-Glo Assay kit (Promega, Madison, WI, USA). For total cholesterol assays, the cells were treated with cholesterol esterase to hydrolyze cholesterol esters to release free cholesterol and fatty acids. The luminescence was measured using a Cytation 5 Cell Imaging Multimode Reader (Agilent/BioTek, Santa Clara, CA, USA).

### 4.4. Generation of FGFR1 Knockout and Stable Expression PCa Cells

The plasmid for FGFR1 knockout (FGFR1^KO^) was generated with Clustered Regularly Interspaced Short Palindromic Repeats (CRISPR)-CRISPR-associated protein 9 (Cas9) system to inactivate the Fgfr1 allele by inserting the single guide RNA (sgRNA; CACATACCAGCTGGATGTCG) into the plasmid LentiCRISPR v2 (Addgene, Watertown, MA, USA). The plasmid carrying the FGFR1 coding sequence was cloned from genomic DNA and inserted into the pWPXL vector (Addgene, Watertown, MA, USA). To prepare Lenti virus carrying these cDNAs, the plasmids were transfected into Lenti-X 293T cells with packaging plasmid psPAX2 (Addgene, Watertown, MA, USA) and pMD2.G (Addgene, Watertown, MA, USA), respectively, together with polyethyleneimine (PEI) (Polysciences, Warrington, PA, USA) to generate the FGFR1^KO^ lentivirus and the FGFR1 overexpression lentivirus. The lentivirus supernatant was collected 48 h post-transfection and stored at −80 °C after filtration with 0.22 µm filters.

To generate DU145 FGFR1^KO^ cells (DU145^R1KO^), we first transduced the cells with the FGFR^KO^ lentivirus with 10 μg/mL polybrene following centrifugation at 1500× *g* for 90 min. After selection with puromycin, the clones of cells were picked up and amplified. To generate FGFR1-rescued DU145 (DU145^R1Res^) cells, the DU145KO cells were further transfected with the FGFR1 lentivirus. To generate LNCaP and C4-2B cells overexpressing FGFR1, designated LNCaP^R1OE^ and C4-2B^R1OE^, respectively, the cells were transduced with lentivirus bearing FGFR1 cDNA. The stably transduced cells were purified by fluorescence-activated cell sorting (FACS) to isolate GFP-positive cells.

### 4.5. Filipin Staining for Free Cholesterol

The cells were seeded in a 4-chamber glass-bottom dish at 0.1 million cells per well in 1 mL complete medium and cultured overnight. The cells were washed with PBS and fixed with 3% paraformaldehyde for 1 h at room temperature. After being rinsed with PBS, the cells were treated with 1.5 mg/mL glycine in PBS for 10 min to quench endogenous fluorescence and stained with 0.05 mg/mL Filipin III (Cayman Chemical, Ann Arbor, MI, USA) for 1 h at room temperature. Cells were again rinsed with PBS and stained with To-PRO-III (Thermo Fisher Scientific, Waltham, MA, USA) for 5 min, with a final wash with PBS. The images were taken with a W1-Yokogawa-Ti2-Nikon Spinning Disk Confocal microscope (Nikon, Lexington, MA, USA) and analyzed by NIS-Elements Viewer (v5.22).

### 4.6. LDL Uptake Assay

The cells were seeded in a 6-well dish at a density of 0.3 million cells per well in 2 mL of complete medium and cultured for 24 h. The cells were starved with serum-free medium overnight and then incubated with 10 μg/mL pHrodo™ Red-labeled LDL (Thermo Fisher Scientific, Waltham, MA, USA) for 2 h. The nucleus was stained with NucBlue™ Live ReadyProbes™ Reagent (Thermo Fisher Scientific, Waltham, MA, USA). The images were captured with a W1-Yokogawa-Ti2-Nikon Spinning Disk Confocal microscope (Nikon, Lexington, MA, USA) and analyzed by NIS-Elements Viewer.

### 4.7. Cholesterol Efflux Assay

The cells were seeded in a 6-well dish at a density of 0.3 million cells per well in 2 mL of complete medium and cultured at 37 °C for 24 h. The cells were starved with serum-free medium overnight. The medium was collected, and the cholesterol levels were measured with the Amplex™ Red Cholesterol Assay Kit (Thermo Fisher Scientific, Waltham, MA, USA). The fluorescence was measured with a Cytation 5 cell imaging multimode reader (Agilent/BioTek, Santa Clara, CA, USA).

### 4.8. Mouse PCa Models

Nude mice and the transgenic adenocarcinoma of the mouse prostate (TRAMP) mice were housed in the Program for Animal Resources (PAR) of the IBT. The principles and procedures are in accordance with the guide from the Institutional Animal Care and Use Committee (IACUC) of TAMU and pre-approved by the IACUC.

For the xenograft assay on nude mice, 1 million PCa cells were suspended in the phosphate-buffered saline (PBS) buffer (GenDEPOT, Baker, TX, USA) and mixed with pre-chilled Matrigel (Corning, Corning, NY, USA) at a 1:1 ratio. A total of 0.1 mL of the cell mixture was injected into the lower right flank of nude mice subcutaneously. Male nude mice of similar age were randomly assigned to experimental groups. Due to the loss of one animal from an unexpected cause, the final group sizes of the WT, KO, and Res groups were *n* = 2, 4, and 3, respectively. One month later, the mice were euthanized in accordance with veterinary guidelines. The tumors were harvested for paraffin embedding for histological and immunostaining analyses.

For the spontaneous tumor formation assay, TRAMP mice were housed for 6 to 8 months, with the tumor closely monitored using a preclinical imaging platform, Vevo 3100 (Fujifilm Visual Sonics, Bothell, WA, USA). Animals were randomly assigned to the control or AZD4547 treatment group, with a final group size of *n* = 3 per group. AZD4547 was administered through intraperitoneal injection at a dosage of 5 μg/g body weight, every two days for 2 weeks. The mice were sacrificed 2 weeks later according to the guidelines. The tumors were harvested for paraffin embedding for histological analyses or homogenized for molecular and biochemical analyses.

### 4.9. Tissue Fixation and Embedding

The tumor tissues collected from the mice were fixed in 4% paraformaldehyde (PFA) overnight. The samples were serially dehydrated in 70% ethanol, 80% ethanol, 90% ethanol, 100% ethanol 1, and 100% ethanol 2 for 1 h at each step. The tissues were then cleared in Xylene 1, Xylene 2, and Xylene 3 for 30 min each, followed by paraffin infiltration in paraffin 1, paraffin 2, and paraffin 3 for 30 min. The tissues were embedded in paraffin and solidified. The samples were stored at 4 °C.

### 4.10. Histology Analysis

The embedded samples were sectioned with a microtome at 5 μm thickness. The sections were deparaffined by incubation in Xylene 1, Xylene 2, and Xylene 3 each for 5 min, and rehydrated in 100% ethanol 1, 100% ethanol 2, 90% ethanol, 80% ethanol, and 70% ethanol each for 2 min. Hematoxylin and Eosin (H&E) staining was performed using the H&E stain kit (Vector LABORATORIES, Newark, CA, USA). After H&E staining, the sections were mounted with Poly-Mount (Polysciences, Inc., Warrington, PA, USA) and sealed with cover slides (VWR, Wayne, PA, USA). The images were taken with a W1-Yokogawa-Ti2-Nikon spinning disk confocal microscope (Nikon). Data were analyzed by the NIS-Elements Viewer software.

### 4.11. Immunofluorescent Staining

The embedded samples were sectioned with a microtome at 5 μm thickness. The sections were deparaffined in Xylene 1, Xylene 2, and Xylene 3 each for 5 min, and rehydrated in 100% ethanol 1, 100% ethanol 2, 90% ethanol, 80% ethanol, and 70% ethanol each for 2 min. The antigen was unmasked in citrate-based antigen unmasking solution (Vector Laboratories, Newark, CA, USA) at 100 °C for 20 min. The slides were blocked with normal horse serum (Vector Laboratories, Newark, CA, USA) and then incubated with primary antibody (listed in Table 1) overnight at 4 °C. The next day, the secondary antibody was incubated at room temperature for 1 h. The images were taken with a W1-Yokogawa-Ti2-Nikon Spinning Disk Confocal microscope (Nikon Lexington, MA, USA). The data were analyzed using NIS-Elements Viewer software.

### 4.12. scRNA-Seq Analysis

The expression matrix was collected from the Gene Expression Omnibus (GEO) database in the National Center for Biotechnology Information (NCBI). The following datasets were used in the assay: GSE137829 [19], GSE176031 [13], GSE172357 [14], GSE153892 [15], GSE181294 [16], and GSE210358 [17]. The tissue collection methods of the publicly available datasets are annotated in the Supplementary Materials (Table S1). The data were analyzed with the Seurat package in RStudio (2024.12.0), and the expression matrix was visualized through UMAP.

Individual cells were manually identified with the signature genes listed below. Markers used for identifying endothelial cells include endoglin (ENG), claudin 5 (CLDN5), von Willebrand factor (VWF), and cadherin 5 (CDH5). Markers used for luminal epithelial cell identification include keratin 8 (KRT8) and keratin 18 (KRT18). Signature genes used to define fibroblast include decorin (DCN), TNF alpha-induced protein 6 (TNFAIP6), apolipoprotein D (APOD), and fibulin 1 (FBLN1). Genes defining basal cells include keratin 5 (KRT5), keratin 14 (KRT14), and tumor protein p63 (TP63). For myeloid cells, the CD14 molecule (CD14), CD68 molecule (CD68), allograft inflammatory factor 1 (AIF1), and colony-stimulating factor 1 receptor (CSF1R) were used for manual cell definition. The ligand–receptor interaction was predicted with CellChat [20].

### 4.13. Bulk RNA-Sequencing

Total RNA samples were collected with the NucleoSpin^®^ RNA Plus kit (TaKaRa). The mRNA was isolated using the NEBNext^®^ Poly(A) mRNA Magnetic Isolation Module (New England Biolabs, Ipswich, MA, USA). The library was constructed using the NEBNext^®^ Ultra™ II Directional RNA Library Prep Kit for Illumina^®^ (New England Biolabs) and the DynaMag™-96 Side Magnet (Invitrogen). The constructed library was purified with the AMPure XP Reagent (Beckman Coulter, Brea, CA, USA). The purified library was quantified by the Qubit 4 Fluorometer (Invitrogen) and the Qubit™ 1× dsDNA High Sensitivity (HS) Assay Kits (Invitrogen). The quality control of the library was performed on a Bioanalyzer Instrument (Agilent, Santa Clara, CA, USA) with the Bioanalyzer High Sensitivity DNA Kit (Agilent) and High Sensitivity DNA Reagents (Agilent). The sequencing was performed on the Next-seq 500 (Illumina, San Diego, CA, USA) at a sequencing read length of 75 bp, single-end. The raw data were analyzed on the Grace platform at the Texas A&M High-Performance Research Computing platform and aligned by Tophat to the GRCh38 reference genome. The expression matrix was analyzed by Cufflinks, and the differentially expressed genes were manually identified by setting fold change larger than 2 or less than −2, with adjusted *p* value less than 0.05. Data visualization, including the volcano plot, the heatmap, the bar plot of gene ontology (GO) analysis, and gene set enrichment analysis (GSEA) were performed in RStudio.

### 4.14. Quantitative Real-Time RT-PCR (qRT-PCR)

Total RNA was collected with the E.Z.N.A total RNA kit I (OMEGA, Norcross, GA, USA). The cDNA was reverse transcribed with the SuperScript™ IV Reverse Transcriptase (Invitrogen). The qRT-PCR was performed on the QuantStudio 6 Flex Real-Time PCR Systems (Applied Bio systems, Carlsbad, CA, USA) using SYBR Green Master Mix (Applied Biosystems, Carlsbad, CA, USA). The data were normalized and displayed as 2^−ΔΔCT^. Primers used are listed in Table 2 and Table 3.

### 4.15. Western Blot Assay

The PCa cells were seeded in a 6-well dish at a density of 0.3 million cells per well in 2 mL of medium. The cells were washed with PBS and then lysed with the radio-immunoprecipitation assay (RIPA) buffer (GenDEPOT, Baker, TX, USA) supplemented with 1% protease inhibitor (GenDEPOT, Baker, TX, USA) and 1% phosphatase inhibitor (GenDEPOT, Baker, TX, USA) on ice. The amount of protein from samples was quantified and balanced with the BCA protein assay kit (Thermo Scientific). All samples were mixed with 5× loading buffer and boiled for 15 min. In total, 10 μg of protein was loaded onto hand-cast SDS-PAGE gels. After electrophoresis, the protein was transferred to polyvinylidene difluoride (PVDF) membranes (Cytiva, Marlborough, MA, USA). The PVDF membranes were blocked with 3% BSA (GenDEPOT, Baker, TX, USA) in TBST, incubated with the primary antibody (Table 3) at a 1:1000 ratio overnight at 4 °C, and then incubated with the secondary antibody at room temperature for an hour. The blot was imaged with the ChemiDoc Imaging System (Bio-Rad, San Francisco, CA, USA).

### 4.16. Statistical Analysis

The statistical significance of gene expression levels measured by the qRT-PCR was determined by an unpaired two-tailed Student’s *t*-test, with a significance threshold set at *p* < 0.05. The analyses were performed using GraphPad Prism (version 10.6.1). Differentially expressed genes (DEGs) from the bulk RNA-seq data were identified using Cuffdiff (v2.2.1), following the Cufflinks RNA-seq workflow [45]. Gene expression levels were quantified by fragments per kilobase of transcript per million mapped reads (FPKM) of each gene in each sample. Raw *p*-values were determined using a Poisson-based statistical model. To correct multiple hypothesis testing, *q*-values were obtained by adjusting *p*-values based on false discovery rate (FDR) using the Benjamini–Hochberg (BH) procedure. DEGs were defined using the threshold of log_2_ (fold change) >1 or <−1 and *q* value < 0.05.

## 5. Conclusions

Collectively, our study reveals a novel mechanism by which FGFR1 elevates cholesterol levels in PCa cells through enhanced uptake and biosynthesis, coupled with reduced efflux. In silico analyses also demonstrate that high expression of FGFR1 is associated with high LDLR expression and clinicopathologic features of human PCa. By integrating data from cellular assays, animal models, and transcriptomic analyses, this work provides new insights into potential therapeutic strategies for advanced PCa. Future studies will be conducted to determine whether modulation of FGFR1 signaling can further enhance the efficacy of immunotherapy in patients with advanced PCa.

## Figures and Tables

**Table 1:** Primary antibodies used for Western blot analysis.

Antibody	Source	Cat#
Anti-FGFR1	CST, Danvers, MA, USA	9740
Anti-phosphorylated-ERK	CST, Danvers, MA, USA	4370
Anti-ERK	CST, Danvers, MA, USA	4695
Anti-β-Actin	Santa Cruz, Dallas, TX, USA	sc-47778
Anti-α-Tubulin	Santa Cruz, Dallas, TX, USA	sc-12462
Anti-LDLR	Santa Cruz, Dallas, TX, USA	sc-18823
Anti-LDLR	Abcam, Waltham, MA, USA	52818
Anti-HMGCR	Invitrogen, Waltham, MA, USA	MA5-31336
Anti-SQLE	CST, Danvers, MA, USA	40659
Anti-HMGCS1	CST, Danvers, MA, USA	42201
Anti-SREBP2	CST, Danvers, MA, USA	25940
Anti-SREBP2	Santa Cruz, Dallas, Texas, USA	sc-271615
Anti-phosphorylated-SREBP2	Invitrogen, Waltham, MA, USA	PA5-106042

**Table 2:** Primer used for qPCR analysis of human FGF gene expression.

Gene	Primer	Sequence, from 5′ to 3′
*FGF1*	Forward	GGAGCGACCAGCACATTCAG
*FGF1*	Reverse	CCGTATAAAAGCCCGTCGGT
*FGF2*	Forward	AAGCGGCTGTACTGCAAAAA
*FGF2*	Reverse	AGTTGTAGCTTGATGTGAGGG
*FGF3*	Forward	GGAGAACAGCGCCTACAGTATT
*FGF3*	Reverse	TGGATCCGCTCCACAAACTC
*FGF4*	Forward	AGCTCTATGGCTCGCCCTTC
*FGF4*	Reverse	ATGCCGGGGTACTTGTAGGA
*FGF5*	Forward	TCTACTGCAGAGTGGGCATC
*FGF5*	Reverse	TCATCTGTGAACTTGGCACTTG
*FGF6*	Forward	AGATTGTACGCAACGCCCAG
*FGF6*	Reverse	GGCAATGTAGGTCCCTTGGT
*FGF7*	Forward	CTCAAGTTGCACCAGGCAGA
*FGF7*	Reverse	CGCTGTTTGCTATTTGACTTTTGT
*FGF8*	Forward	GGGTGTCTCCCAACAGGTAAC
*FGF8*	Reverse	CCGTCTCCACGATGAGCTTT
*FGF9*	Forward	TTTGGGAATGTGCCCGTGT
*FGF9*	Reverse	AATTCCAGAATGCCAAATCGG
*FGF10*	Forward	GAGAACTGCCCGTACAGCATC
*FGF10*	Reverse	GCCTCCCATTATGCTGCCA
*FGF16*	Forward	GAGATCTTCCCCAACGGCAC
*FGF16*	Reverse	CGTGTGAGTTTCTTCGACCCAT
*FGF17*	Forward	CTGAGGAACCTCTCCAGCGA
*FGF17*	Reverse	GGGTGATTCTCCCCCTGAGTT
*FGF18*	Forward	TGCTTCCAGGTACAGGTGCT
*FGF18*	Reverse	ATGTGTTTCCCACTGGTCCG
*FGF19*	Forward	CAGAGCGCGCACAGTTTG
*FGF19*	Reverse	GCGGATCTCCTCCTCGAAAG
*FGF20*	Forward	GACCACAGCCTCTTCGGTATC
*FGF20*	Reverse	GTGCCACAAAATACCTGCGG
*FGF21*	Forward	GAGCCATTGATGGACTCGGA
*FGF21*	Reverse	TGCAGGAGACTTTCGGGG
*FGF22*	Forward	CACGGCCAGGACAGCATC
*FGF22*	Reverse	CCTGCAGTCCACGGTGTAG
*FGF23*	Forward	AGAGGATGCTGGCTTTGTGG
*FGF23*	Reverse	TCCGGGTCGAAATAGTGTGAT

**Table 3:** Primer used for qPCR analysis of human cholesterol homeostasis gene expression.

Gene	Primer	Sequence, from 5′ to 3′
*LDLR*	Forward	ACTGTCCGAAGCCTGTTCTG
*LDLR*	Reverse	AGCTACCCCTCGAGACAGAT
*HMGCR*	Forward	GTGAGATCTGGAGGATCCAAG
*HMGCR*	Reverse	CCCCACTATGACTTCCCAGG
*HMGCS1*	Forward	CGGACTGTCCTTTCGTGGC
*HMGCS1*	Reverse	TTCCTCCTTCGGGCACAAGC
*FDFT1*	Forward	ACCTACTCCACAGGTCCAGC
*FDFT1*	Reverse	CTGCTGAGCGAGTCCTGGTC
*SQLE*	Forward	GCCTGCCTTTCATTGGCTTC
*SQLE*	Reverse	TTCCTTTTCTGCGCCTCCTG
*LSS*	Forward	GAGCGGCGTTATTTGCAGAG
*LSS*	Reverse	CCCCAGCAATGTTTTCCTGC
*DHCR24*	Forward	GACCTCCATTGGCTGGACTC
*DHCR24*	Reverse	GGTCTGAGTTTTCGGACGGA
*ABCG1*	Forward	TGTCTGATGGCCGCTTTCTC
*ABCG1*	Reverse	TCAGGAGGGTCTTGTATCCTTTC
*ABCA1*	Forward	CAGAGGTGGCTCTGATGACC
*ABCA1*	Reverse	TAGCACAGGCAGATTGGTGG
*ACTB*	Forward	ACAGAGCCTCGCCTTTGCC
*ACTB*	Reverse	GATATCATCATCCATGGTGAGCTGG

## Data Availability

The original contributions presented in this study are included in the article/Supplementary Materials. Further inquiries can be directed to the corresponding author F.W.

## Supplementary Materials

The following supporting information can be downloaded at: https://www.mdpi.com/article/10.3390/ijms27031190/s1.
