# Supplementary material for: Ectopic FGFR1 Increases Intracellular Pool of Cholesterol in Prostate Cancer Cells
Source: Int J Mol Sci. 2026 Jan 24;27(3):1190. doi: 10.3390/ijms27031190 (PMC12898303; doi:10.3390/ijms27031190)
Supplement: Supplementary file 1 [file ijms-27-01190-s001.zip › ijms-4043520-supplementary.pdf]

Supplemental Table S1. Sample resources for single-cell RNA-sequencing analysis.

Datasets	Tissue collection methods
GSE137829	Prostate biopsies
GSE176031	Biopsies, radical prostatectomies,
GSE172357	Organ donors
GSE153892	Unidentified
GSE181294	Invasive transabdominal radical prostatectomy.
GSE210358	Radiologically guided biopsies
